# Developmental Characterization of the MicroRNA-Specific *C. elegans* Argonautes *alg-1* and *alg-2*

**DOI:** 10.1371/journal.pone.0033750

**Published:** 2012-03-20

**Authors:** Alejandro Vasquez-Rifo, Guillaume Jannot, Javier Armisen, Michel Labouesse, Syed Irfan Ahmad Bukhari, Evelyne L. Rondeau, Eric A. Miska, Martin J. Simard

**Affiliations:** 1 Laval University Cancer Research Centre, Hôtel-Dieu de Québec (CHUQ), Quebec City, Québec, Canada; 2 Wellcome Trust Cancer Research UK Gurdon Institute, The Henry Wellcome Building of Cancer and Developmental Biology, University of Cambridge, Cambridge, United Kingdom; 3 Department of Biochemistry, University of Cambridge, Cambridge, United Kingdom; 4 Development and Stem Cells Program, IGBMC, CNRS (UMR7104), INSERM (U964), Université de Strasbourg, BP10142, Illkirch, France; Brown University, United States of America

## Abstract

The genes *alg-1* and *alg-2* (referred to as “*alg-1/2*”) encode the Argonaute proteins affiliated to the microRNA (miRNA) pathway in *C. elegans*. Bound to miRNAs they form the effector complex that effects post-transcriptional gene silencing. In order to define biological features important to understand the mode of action of these Argonautes, we characterize aspects of these genes during development. We establish that *alg-1/2* display an overlapping spatio-temporal expression profile and shared association to a miRNAs set, but with gene-specific predominant expression in various cells and increased relative association to defined miRNAs. Congruent with their spatio-temporal coincidence and regardless of *alg-1/2* drastic post-embryonic differences, only loss of both genes leads to embryonic lethality. Embryos without zygotic *alg-1/2* predominantly arrest during the morphogenetic process of elongation with defects in the epidermal-muscle attachment structures. Altogether our results highlight similarities and specificities of the *alg-1/2* likely to be explained at different cellular and molecular levels.

## Introduction

Argonaute family proteins are defined by the presence of the PAZ domain which contributes to the binding of small (21–32 nucleotide long) RNA molecules and the PIWI domain that confers the endonuclease enzymatic activity present in some members of the Argonautes family [1]. This protein family is conserved from archaea to eukarya, and largely expanded in some plants and animals. Distinct kinds of small RNAs are bound by Argonautes which participate in viral defence [2], post-transcriptional gene regulation [3] and transposon silencing [4], [5], processes which in turn affect somatic and germline development.

In the nematode *C. elegans*, the Argonaute family comprises 24 genes and 2 pseudogenes, which partition into three groups, the Argonaute-like (AGO-like), the Piwi-like and a nematode-specific clade [1]. The genes *alg-1* and *alg-2* (hereafter, both referred to as “*alg-1/2*”) belong to the conserved AGO-like clade, which includes the fly *D. melanogaster* Ago1 and Ago2, and four mammalian Argonautes, AGO1-4. *Alg-1/2* have been shown to be required for the miRNA pathway but not for exo-RNAi [6], while the fly and mammalian AGOs are differentially required for both RNAi and the miRNA pathway [7], [8], [9]. Two additional *C. elegans* Argonautes in the same clade, *alg-3* and *alg-4*, are required for the accumulation of 26-nt long endogenous RNAs and affiliate to a separate endo-RNAi pathway [10].

The canonical miRNA pathway involves the processing of a primary RNA molecule by the RNase III enzyme Drosha, to produce a stem-loop precursor molecule which is next cleaved by the Dicer enzyme to release a small double-stranded RNA moiety (21–23 nt). One of strands from this small duplex will then be loaded into the Argonaute protein, forming the core complex that targets the 3′ untranslated region (UTR) of mRNAs with sequence specificity to elicit post-transcriptional gene silencing [3].

In distinct organisms, multiple Argonautes are involved in the miRNA pathway. In mammals, ectopic expression of the AGO1-4 Argonautes is able to provide miRNA function in cells [8], while fly Ago1 is typically loaded with most miRNAs, some specific ones are bound by Ago2 [9], [11]. The presence of several Argonautes in the miRNA pathway implies possible redundant and specialized functions, an aspect still incompletely understood. In this report, we describe the expression and embryonic phenotypes of *C. elegans alg-1/2* along with their post-embryonic miRNA interaction profiles with the purpose of providing insights into the shared and the non-redundant functions of the Argonautes of the miRNA pathway.

## Results

### Structural features of ALG-1 and ALG-2

**Figure 1:**
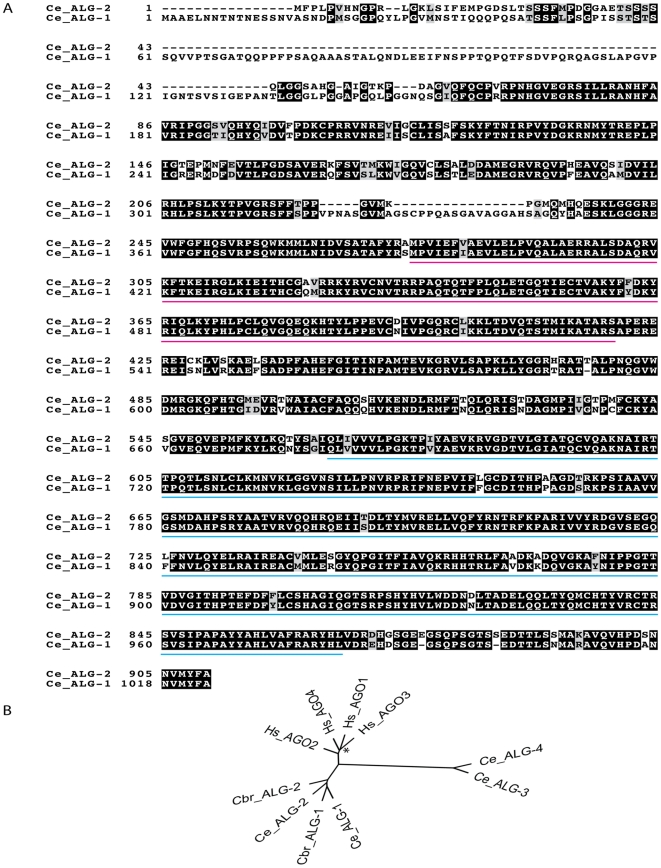
The *C. elegans* microRNA Argonautes *alg-1* and *alg-2*. (**A**) Amino acid sequence alignment between ALG-1 and ALG-2 proteins (77% identity and 81% similarity). Identical (black) and similar (grey) residues as well as the PAZ (pink) and PIWI (blue) signature domains are indicated. (**B**) Neighbor joining tree of nematodes and human AGO-clade Argonautes based on the conserved sites from an alignment of full length protein sequences. With the exception of human AGO1 and AGO3 (*), all the subtrees are robustly significant (higher than 95% bootstrap; 500 trials). Ce, *Caenorhabditis elegans*; Cb, *Caenorhabditis briggsae*; Hs, *Homo sapiens*.

The *C. elegans* genes *alg-1* and *alg-2* encode two Argonaute proteins with high amino acid sequence similarity (81%; Fig. 1A). The PAZ, PIWI and C-terminal regions of the ALG-1/2 are highly conserved, while the N-terminal region is constituted of amino acids specific to each Argonaute, especially prominent in ALG-1. The N-terminus is involved in protein interactions which confer specificity to ALG-1 [12].

The highly conserved orthologs of both genes that are readily identified in other *Caenorhabditis* species (Fig. 1B) along with the high *alg-1* and *alg-2* DNA sequence similarity, indicates that these genes most probably arose by recent gene duplication as previously suggested by Grishok and collaborators [6].

### Expression pattern of ALG-1 and ALG-2

**Figure 2:**
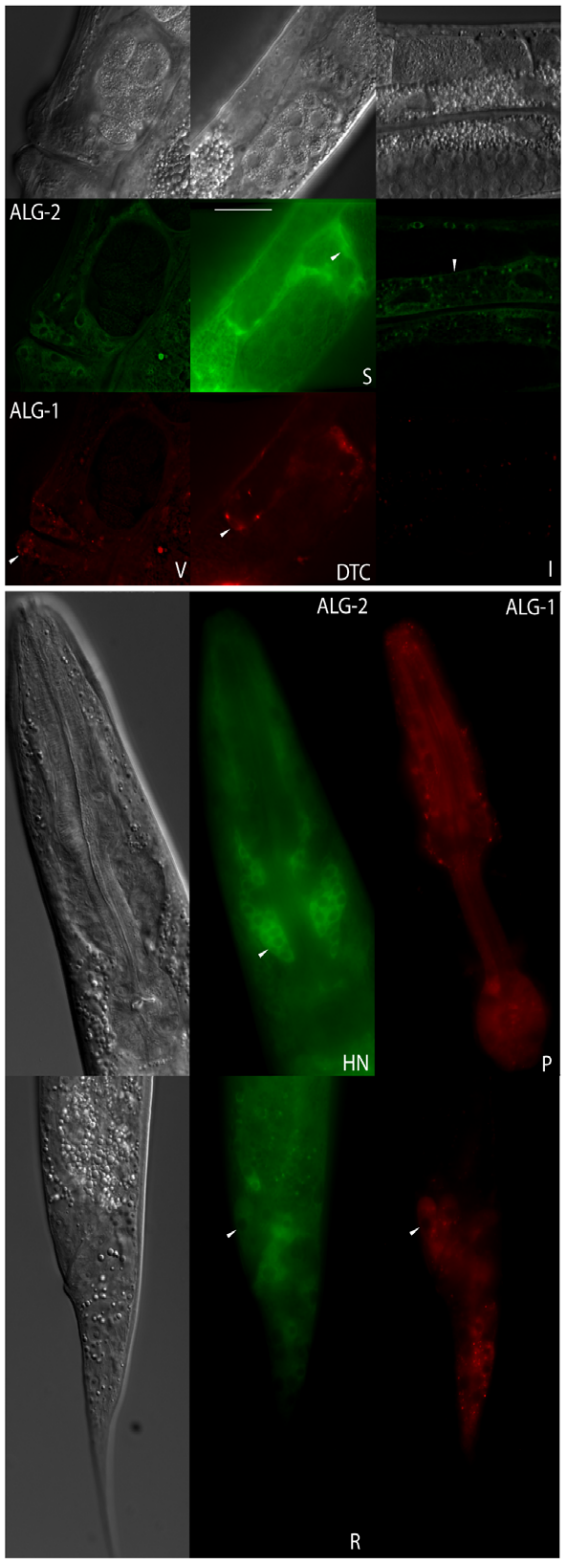
ALG-1 and ALG-2 expression profile in adult worms. **Top panel:** GFP::ALG-2 and RFP::ALG-1 are co-expressed in most tissues including vulva (V) and spermatheca (S). **Bottom panel:** Predominant GFP::ALG-2 expression is seen in a set of head neurons (HN) and tail cells (T) while RFP::ALG-1 is strongly expressed in the pharynx (P) and some tail cells (T). Scale bar 20 µm.

In order to investigate functional differences between *alg-1* and *alg-2*, we first examined their expression pattern using functional translational reporters containing both ALG-1 and ALG-2 tagged with GFP or RFP, preserving their endogenous promoters and UTRs in the respective mutant backgrounds. For simplicity, we refer to the GFP::ALG-2 and RFP::ALG-1 reporters as ALG-2 and ALG-1, respectively. Both Argonautes were found to be broadly expressed in most tissues, but their expression patterns were not completely overlapping. Subsets of neurons in the head ganglia expressed predominantly ALG-2, while the pharynx more prominently expressed ALG-1 (Fig. 2). Cells in the tail also displayed specific expression. Nonetheless, both Argonautes are expressed together in tissues including the vulva, seam cells, ventral nerve chord and somatic gonad (Fig. 2, Table 1, Fig. S1 and Fig. S2). Identical ALG-1 expression was also observed for the common tissues examined by Chan and Slack using similar translational reporter [13]. To confirm that the expression pattern observed with chromosomal arrays reflects the expression of endogenous protein, we performed a whole-worm immunostainings using a polyclonal antibody raised against the ALG-1 specific N-terminus region (ALG-2 specific antibody is not yet available). The endogenous ALG-1 expression was confirmed for the pharynx and head neurons (Fig. S3) further supporting the expression pattern observed with transgenic lines.

**Table 1:** Summary of ALG-1 and ALG-2 expression in different organs and tissues.

Tissue	Abbrev.	ALG-1	ALG-2
Intestine	I	+	+
Pharynx	P	+	−
Head Hypodermis	HH	+	+
Body Hypodermis	BH	+	+
Tail Hypodermis	TH	+	+
Seam cells	SC	+	+
Excretory system	X	+	+
Spermatheca	S	+	+
Distal Tip Cells	DTC	+	+
Uterus	U	+	+
Gonadal Sheath	GS	+	+
Vulva	V	+	+
Body Neurons	BN	+	+
Head Neurons	HN	−	+
Tail Neurons	TN	+	+
Body Muscle	BM	+	+
Rectum	R	+	+

Fluorescence intensity is indicated as + to indicate a discernible signal, and – if signal was not clearly discernible from background fluorescence.

**Figure 3:**
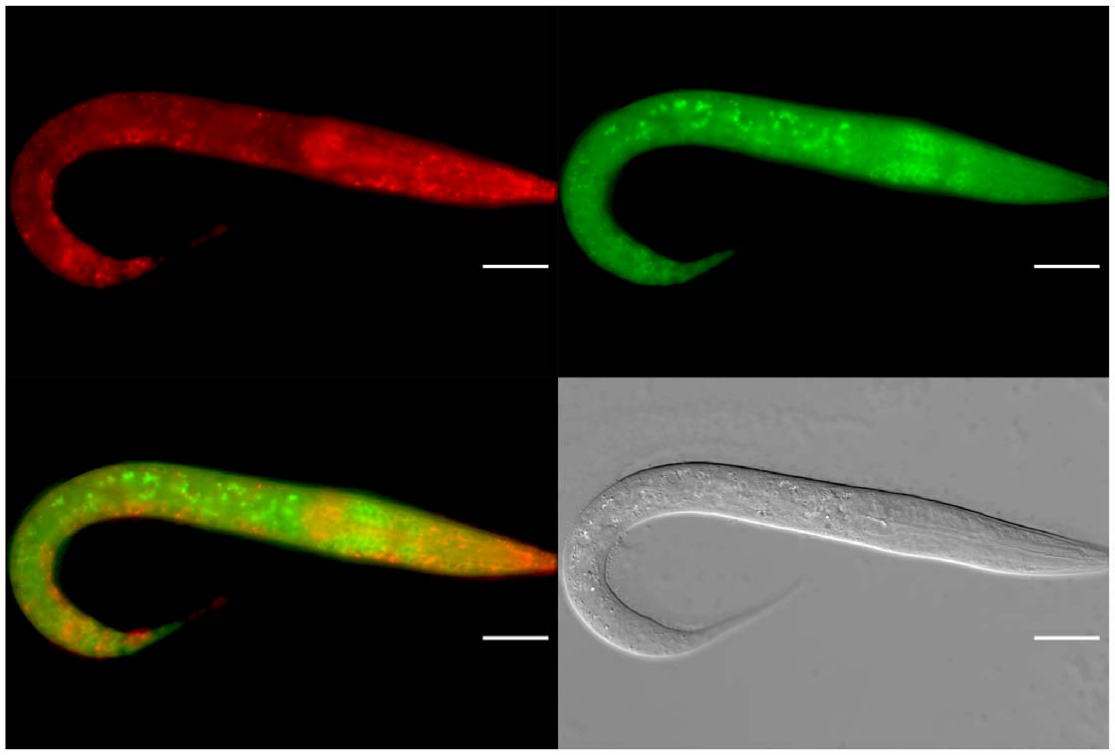
Profile of ALG-1 and ALG-2 expression in larval stage. Expression profile of GFP::ALG-2 and RFP::ALG-1 as seen in the L1 larval stage. Subsequent larval stages (L2 to L4) and adults display the same expression profile (not shown). Scale bar 20 µm.

**Figure 4:**
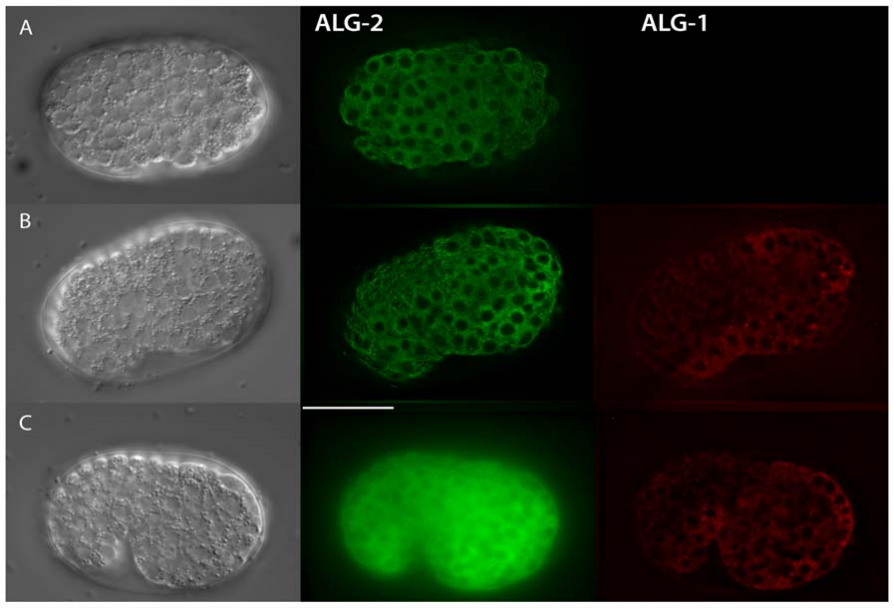
Embryonic ALG-1 and ALG-2 expression. The expression onset of GFP::ALG-2 and RFP::ALG-1 differs. RFP::ALG-1 fluorescence is first detected at the beginning of the morphogenetic phase (B). Scale bar 20 µm.

We next examined the ALG-1/2 expression in distinct *C. elegans* stages. Examination of the ALG-1/2 expression during larval development did not reveal differences in expression during the four larval stages and adults (Fig. 3). However, during embryogenesis the onset of zygotic expression was distinct. ALG-2 started to be expressed from pre-morphogenetic embryonic stages while ALG-1 is first detected at the beginning of the morphogenetic phase (Fig. 4). Altogether, our data indicated that the ALG-1/2 expression patterns are spatially and temporally overlapping with some specific tissues where one of the Argonautes is predominantly expressed.

### microRNA interactions with ALG-1 and ALG-2

**Figure 5:**
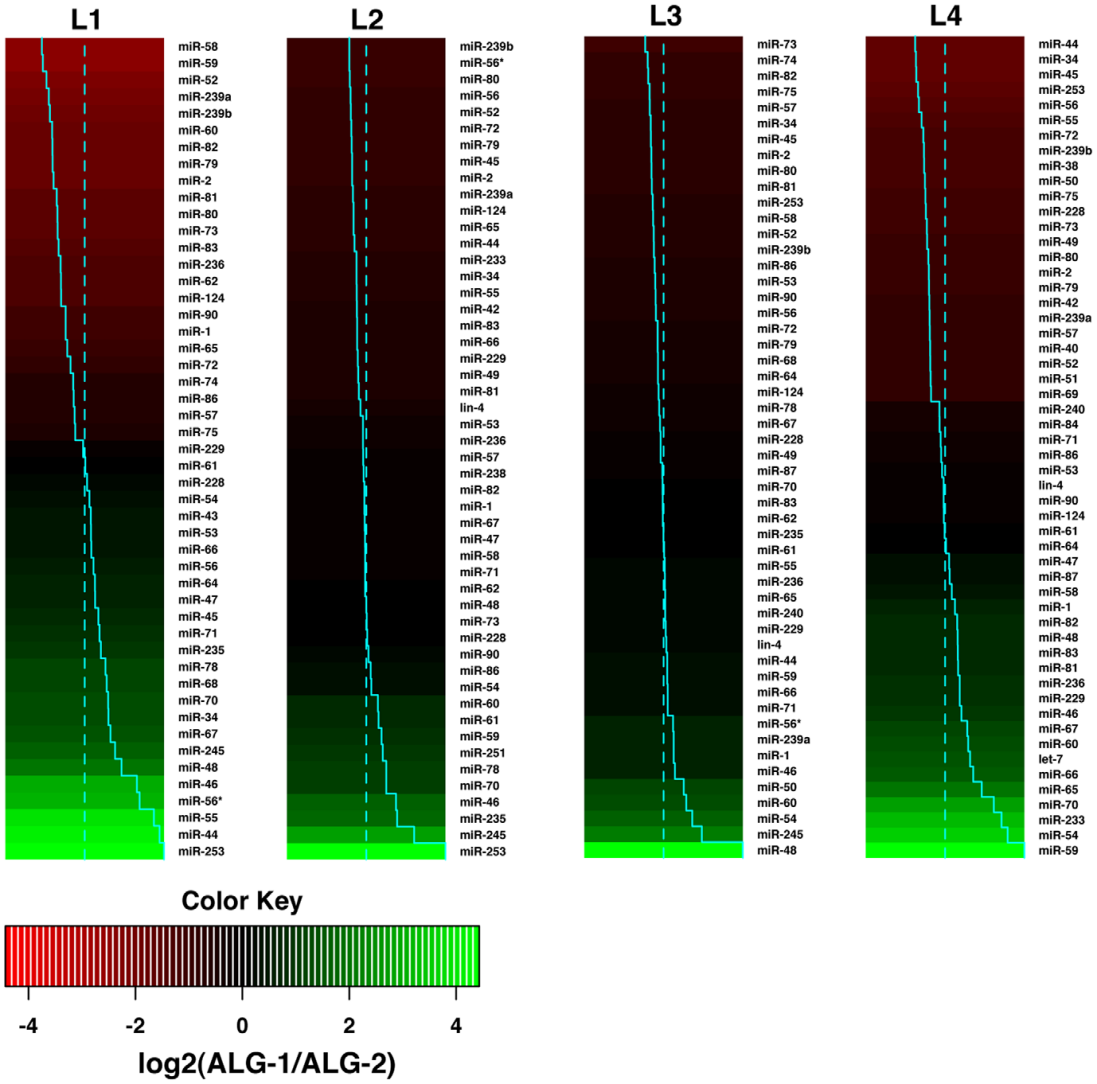
Relative microRNA association with ALG-1 and ALG-2. Heatmaps representing the ratios of ALG-1 to ALG-2 associated miRNAs at the larval stages indicated as estimated from miRNA microarrays (towards red: stronger association to ALG-2, towards green: stronger association to ALG-1). miRNA expression data were filtered for robustly expressed miRNAs. Ratios were log2 transformed, centered and normalized for each column. The distance of the solid blue line from the center of each color-cell is proportional to the ratio. Mean ratio indicated as dotted blue line.

To complement the ALG-1/2 expression patterns, we studied the association of a set of miRNAs with ALG-1 and ALG-2 throughout the four larval stages (L1–L4). To achieve this, we first immunoprecipitated ALG-1 and ALG-2 complexes from synchronized larval populations of transgenic worms expressing either ALG-1 or GFP::ALG-2 functional integrated transgenes (Fig. S4). We next purified small RNAs from ALG-1/2 complexes and determined miRNA association by microarrays. While control immunoprecipitations do not show significant difference in miRNA association (data not shown), at each stage of development, we detected a number of miRNAs associated preferentially to ALG-1 or ALG-2 and notably this preferential association was higher in the L1 and L4 stages compared to the L2/L3 stages (Fig. 5). The relative association of miRNAs to the Argonautes at each developmental stage followed a trend of conservation at other stages, but for some miRNAs it changed dramatically during development (*e.g.* miR-44 in L1 and L3 stages associates more with ALG-1 while at L2 and L4 it does with ALG-2, miR-253 switched its relative association from the L1/L2 stages to L3/L4). Therefore, our analysis indicates that while ALG-1/2 largely bind to the same set of miRNAs, the association of specific miRNAs to either Argonaute supports the existence of specificity at the molecular or cellular level that follows specific dynamics during development.

### ALG-1/2 are required for embryonic morphogenesis

Although *alg-1* and *alg-2* are highly similar at the sequence level and have mostly overlapping expression patterns, mutants of these Argonautes differ substantially in their phenotypes. Comparison of putative null alleles, reveals that *alg-1(gk214)* mutants have lesser growth and fertility compared to *alg-2(ok304)* [14] and much more penetrant miRNA-related phenotypes like gapped alae (*alg-1* 24%, *alg-2* 0%, n = 60) and bursting through the vulva (*alg-1* 27%, *alg-2* 0%, n = 40). Besides these post-embryonic phenotypes, neither *alg-1(gk214)* or *alg-2(ok304)* have detectable embryonic lethality under standard growth conditions. To further precise the two Argonautes embryonic phenotypes, a balanced strain with deletion alleles of both genes was constructed (*alg-2(ok304)*; *alg-1(gk214)*/*unc-84(e1410)*). The strain segregated the expected genotypes but no homozygous *alg-2(ok304)*; *alg-1(gk214)* double mutants (hereafter referred to as double mutants) were ever found as viable worms. Indeed, homozygous double mutants arrested as embryos, consistent with the phenotype observed in simultaneous RNAi knockdown of *alg- 1* and *alg-2* [6].

**Figure 6:**
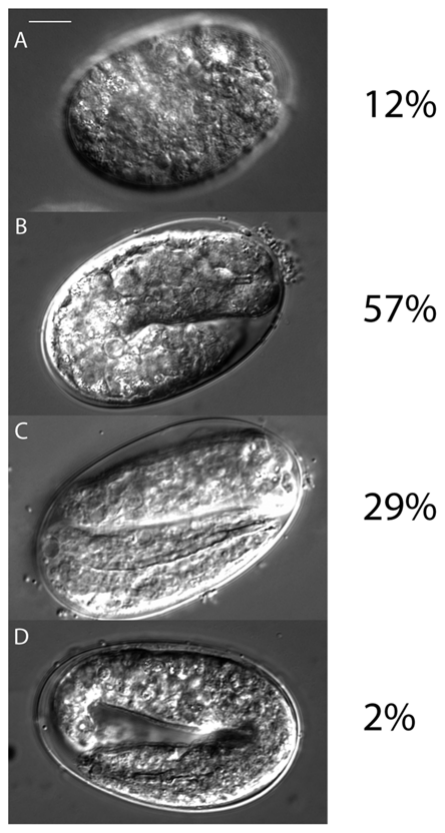
ALG-1 and ALG-2 are required for *C. elegans* embryonic development. Statistics of embryonic arrest at 15°C (n = 272). Freshly layed eggs on petri dishes were examined after a period of 12 h. A major fraction of double mutant embryos arrest during the morphogenetic phase of development (**B–D**), mainly at the 2-fold stage (**B**).

The embryonic arrest of the double mutant indicates that at least one of the two Argonautes has to be zygotically expressed in the embryo to allow complete development. The double mutant predominantly arrested at the 2-fold stage of development at 15 and 25°C (Fig. 6 and data not shown). This stage is part of the morphogenetic phase of development, which follows after most embryonic cell divisions have taken place and comprises major shape changes and completion of organogenesis. The fraction of embryos that arrested either after complete elongation or before morphogenesis is possibly due to incomplete penetrance and variation of the maternally contributed ALG-1 and ALG-2.

**Figure 7:**
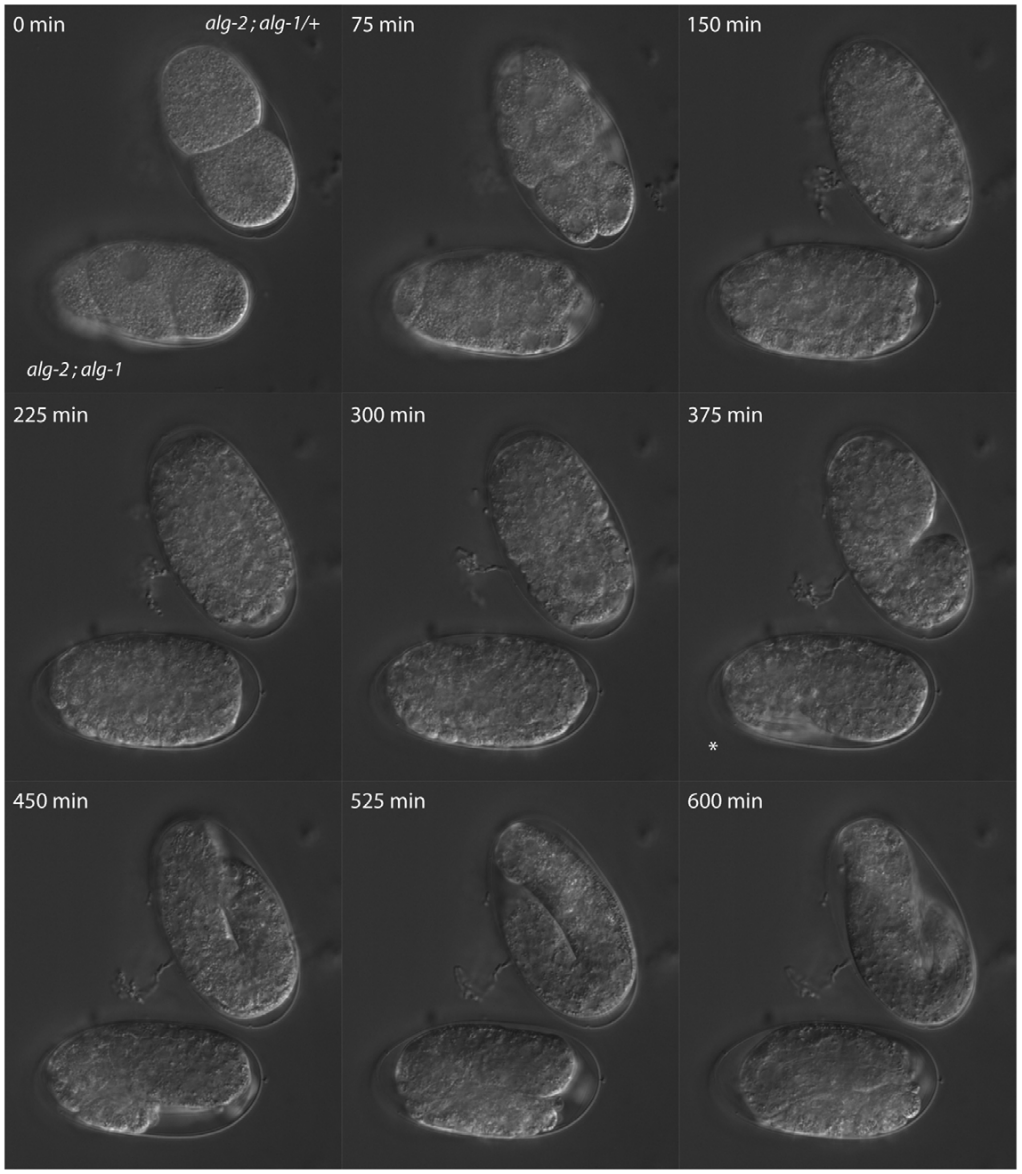
Developmental timing of double *alg-1/2* mutant and siblings. Time-lapse microscopy of *C. elegans* embryos. Both embryos proceed through development at similar rates until the morphogenetic phase (375 min) were the *alg-2(ok304); alg-1(gk214)* double mutant arrest (asterisk). Viable siblings were able to proceed development normally. The double mutant embryos are not paralyzed and are able to twitch (see Movie S1).

To further characterize the embryonic requirement of *alg-1/2*, time-lapse microscopic recordings were conducted. We did not detect developmental defects in the double mutants before the 2-fold stage, and the recordings revealed no evident difference in developmental timing between the double mutant embryos and their viable siblings prior to the 2-fold stage were arrest occurred (Fig. 7 and Movie S1). This supports that the 2-fold arrest is likely caused by the disruption of one or various morphogenetic processes taking place and not the consequence of an earlier embryonic defect.

### ALG-1 and ALG-2 requirement in epidermal cytoskeleton and muscle development

**Figure 8:**
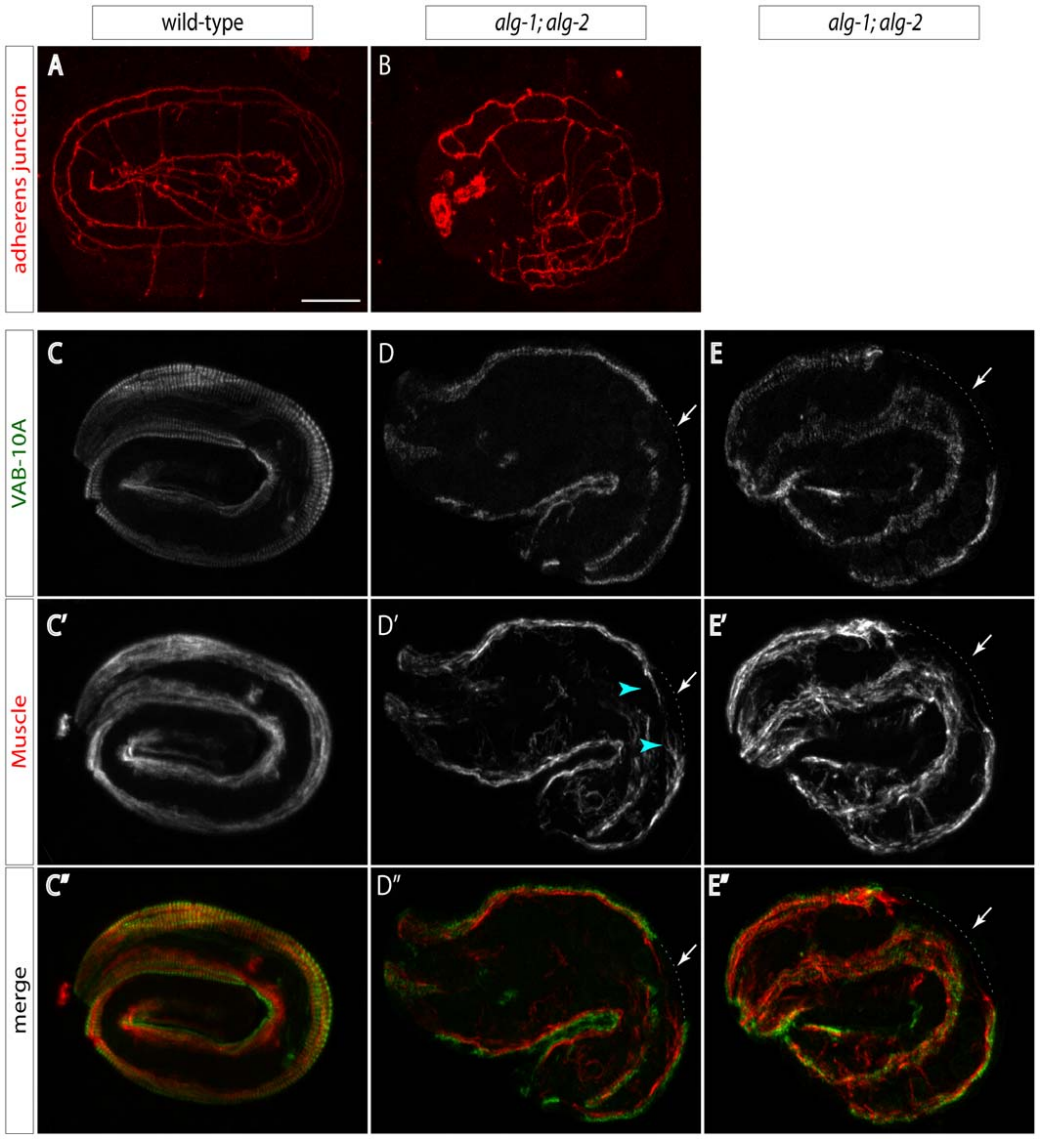
ALG-1/2 function is required to maintain muscles and the epidermis. (**A–B**) Embryos collected between 6–8 hours post egg-laying at 25°C and stained with the antibodies MH27 (adherens junction). The *alg-1; alg-2* mutant embryo did not progress beyond the two-fold stage, yet has grossly normal junctions. (**C–E**) Embryos collected between 6–8 hours post egg-laying at 25°C and stained with the antibodies 4F2 (VAB-10A; C–E) and NE8/4C6 (muscle; C′–E′); merge picture (C″–E″). Arrow, are where the fibrous organelle (D–E) and muscle (D′–E′) pattern is partially interrupted; in addition, muscles do not closely follow the body wall in this area (they should be closer to the blue dotted line; see blue arrowheads). Embryos did not elongate beyond the two-fold stage.

The observed embryonic arrest may reflect an impairment of one or several of the process taking place during the morphogenetic phase of development, namely epidermal migration, ventral enclosure and elongation [15]. During this phase, the epidermal cells, initially located in the embryonic dorsal part, intercalate and extend around the embryo towards the ventral side and fully enclose it. Upon completion of epidermal enclosure, embryos continue to elongate anteroposteriorly, a process whereby epidermal and muscle cells actively drive the constriction of the embryo along its cross-section. Examination of the epidermal adherens junctions, as judged by staining with the junction-specific monoclonal antibody MH27, did not reveal any apparent epithelial polarity defect and revealed a minor epidermal cell shape defect commonly found in embryos with partial or severe muscle defects (data not shown). Consistent with DIC pictures (Fig. 6), embryos could not elongate much beyond the two-fold stage resulting in slightly deformed embryos (Fig. 8B). Thus, proper epidermal specification, dorsal intercalation and ventral enclosure are achieved in the double mutant embryos with very few exceptions.

The elongation process depends on the concerted epidermal and muscle function, and both tissues connect through specialized junctions containing hemidesmosomes and intermediate filaments (known as fibrous organelles) [16]. To investigate their integrity, we co-stained embryos from *alg-2(ok304)*; *alg-1(gk214)*/*unc-84(e1410)* parents with a hemidesmosome marker (antibodies against VAB-10A protein) and a monoclonal antibody against a muscle marker (NE8/4C6). We observed that fibrous organelles were interrupted, and that muscles appeared to detach from the body wall in those areas in most putative *alg-2(ok304)*; *alg-1(gk214)* embryos: out of 80 embryos laid by heterozygous parents, 21 had elongation defects (putative double mutants), 18 of which also showed fibrous organelle and/or muscle defects (Fig. 8). However, fibrous organelle defects were not as severe as in very strong fibrous organelle mutants such as *vab-10* and *vab-19*, or in *vab-10(e698); eel-1(ok1575)* double mutants [17]. The muscle NE8/4C6 staining pattern was also less severe than in the most severe *pat* mutants which disrupt the attachment of myofibers to the muscle plasma membrane [18].

Altogether our immunofluorescence data are consistent with the time-lapse analysis and suggest that defective embryonic morphogenesis is the primary cause of lethality of the double mutant. The penetrant fibrous organelle defect is compatible with either disruption of a fibrous organelle component, or of a muscle component.

## Discussion

Our study indicates that the Argonaute encoding *alg-1* and *alg-2* genes have remarkable similarities that do not preclude major functional differences. Multiple resemblances include high sequence homology, overlapping expression patterns, partially coincident sets of bound miRNAs, and their redundant requirement for embryonic development. However, single mutant of these genes lead to very distinct phenotypes and effects on the miRNA pathway during worm post-embryonic stages. The more penetrant defects seen in *alg-1* mutants do not correlate with differences in the relative abundance of ALG-1/2, as judged by western analysis using antibody against the conserved region of ALG-1/2 ([19]; unpublished observations).

The detailed analysis of the expression pattern of *alg-1* and *alg-2* demonstrates that ALG-1/2 proteins are overlapping in many tissues, but a specific expression pattern is observed in a subset of head neurons, pharynx and specific cells in the tail. The embryonic onset of expression differs and leads to a pattern that remained constant from the first larval stage to adults. The differences in the *alg-1/2* expression patterns may result in part from transcriptional differences. Genome-wide analysis of the transcription factor PHA-4 has found that it binds to the *alg-1* promoter, while no significant binding is detected in the promoter (2 kb upstream from start site) of *alg-2* [20]. Thus, PHA-4 and additional transcription factors may contribute to the specificity of the *alg-1/2* expression in certain tissues.

In agreement with the observed expression overlap, miRNA profiling during the worm larval stages showed that ALG-1/2 bind the studied set of miRNAs coincidently although a preferential association is detected for some miRNAs. Similar miRNA-Argonaute associations have also been observed for human Argonautes in cultured cells [21]. This preferential association could arise in two ways, differential co-expression of Argonaute and miRNA in certain cells, or as result of molecular Argonaute-miRNA specificity. It should be noticed that observed preferential association of some miRNAs to ALG-1/2, does not constitute an exclusive association that would make specific miRNAs completely dependent on the presence of either ALG-1 or ALG-2.

In a first scenario, the co-expression of a specific Argonaute and miRNA could increase their relative association. The expression of many *C. elegans* miRNAs has been described [22], [23], [24] and ranges from highly specialized (*i.e. lsy-6*) to widely expressed. The presence of specific tissues where one of the *alg-1/2* is predominantly expressed, and the specialized expression seen in some miRNAs could dictate specific miRNA-ALG-1/2 interactions in certain cells or tissues. The correlation of miRNA and ALG-1/2 expression is subject to confounding effects given that the relative association we measured reflects the contribution of all the tissues were the miRNA is expressed. In few cases a correlation is straightforward, ALG-2 is predominantly expressed in neurons of the head ganglia, and it associates preferentially with the miR-72 miRNA that is expressed only in the head neurons [22].

Alternatively, molecular specificity may explain preferential miRNA-Argonaute association. The identity of the 5′ terminal nucleotide confers affinity on small RNAs for different plant Argonautes [25], and duplex mismatches sort out siRNA and miRNA in flies and *C. elegans* [26], [27], [28]. However, it is yet unknown what could determine the specificity of Argonaute proteins for particular miRNAs. The sequence and structure of miRNAs and their precursors molecules as well as Argonautes interactions with additional factors could potentially confer this specificity. This kind of features may potentially explain the relative association observed among ubiquitously expressed miRNAs such as miR-52 and miR-71 that are associated preferentially with ALG-2 and ALG-1, respectively.

Our observations also demonstrate that genetically, *alg-1/2* share functions during embryonic development. The embryonic lethality of the *alg-1/2* double mutant is not observed in single mutants of Dicer and Drosha, due to the fact that the maternal contribution of these enzymes of the microRNA pathway allows homozygous mutants to complete all developmental stages and become sterile adults [29], [30]. In contrast, the embryonic lethality of the double mutant reveals that *alg-1/2* maternal contribution is insufficient to complete development, and thus zygotic expression is required. The *alg-1/2* maternal contribution [31] present in the double mutant, disallowed the examination of the ALG-1/2 role during early development. An early developmental role for specific miRNAs and associated Argonautes in the degradation of maternal transcripts at the maternal to zygotic transition has been documented in zebrafish [32] and the *X. laevis* [33], and could be explored in *C. elegans* by using RNAi. Considering that *alg-1/2* function in the miRNA pathway and that most miRNAs are not essential for development [34], [35], the observed embryonic arrest may be the result of the combined loss of a specific set of miRNAs. To date, the absence of the complete *mir-35* and *mir-52* families has been reported as embryonic lethal. The loss of miR-35 family causes a slower development and arrest at the 2-fold to 3-fold stage [35], while the miR-52 family mutant displays unattachment of the pharynx at a late stage of embryonic development [36]. Identification of the miRNAs and targeted genes involved in the embryonic arrest remains open for future work.

Beside *C. elegans*, mutants of the miRNA Argonautes in mammals and *Drosophila* also display embryonic lethality. Ago2 knockout mice display early mesoderm defects [37], [38], as well as mid-gestational death due to placental defects [7], [39]. Similarly, ago1 and ago2 are together essential for the establishment of segment polarity in flies [40]. Some of these essential developmental roles of the miRNA pathway during animal development may reflect a common requirement of the pathway for the proper differentiation and function of certain tissues. Along this line, our data demonstrate the requirement of *alg-1/2* during the morphogenetic phase of embryonic development, manifested through the predominant arrest at the 2-fold stage, where worms are unable to complete the elongation process. For the most part, the epidermal and muscle tissues of the double mutant embryos are properly specified and epidermal adherens junctions presumably normal. In contrast, the epidermal-muscle attachment structures are mildly but frequently affected. The defects most likely stem from epidermal fibrous organelle or muscle defects. Since both the elongation defects and fibrous organelle staining defects were less severe than those observed in core fibrous organelle mutants, we do not think that ALG-1 and ALG-2, hence that miRNAs, are essential for the production of a core fibrous organelle component. We recently established that muscle contractions are required to pattern hemidesmosomes and promote epidermal morphogenesis [41]. Hence, one or more miRNAs might contribute either to some aspect of muscle differentiation and/or contractility, or within the epidermis to relay the muscle-to-epidermis mechanical signal, possibly in a feedback loop. Future experiments should help clarify among those possibilities.

## Materials and Methods

### Culture conditions and general methods

Worms were cultured in standard conditions [42]. All experiments were performed at 20°C unless otherwise noted.

### Transgenic strains

The following transgenic strains were generated by microinjecting mix of plasmids and UV integrated as described in [43], [44]:

MJS13: *alg-1(gk214)* In[*alg-1p*::*rfp::alg-1::alg-1 3′UTR* ; *alg-2p::gfp::alg-2::alg-2 3′UTR* ; pRF4]
MJS18: *alg-2(ok304)* In[*alg-1p::gfp::alg-1::alg-1 3′UTR* ; *alg-2p::rfp::alg-2::alg-2 3′UTR* ; pRF4]
MJS46: *alg-1(gk214)* In[*alg-1p::alg-::alg-1 3′UTR* ; pRF4]
MJS26: *alg-2(ok304)* In[*alg-2p::gfp::alg-2::alg-2 3′UTR* ; pRF4]

### Microscopy

Worms were examined mounted on agar pads using a Zeiss axioimager M1 microscope.

Time-lapse recordings were done using the AxioVision (Release 4.8) software at 1 minute interval during 600 minutes.

### Immunostainings

For embryo stainings, heterozygous *alg-2*(ok304); *alg-1*(gk214)/*unc-84*(e1410) mothers were propagated at 25°C (non-permissive for *unc-84*) and allowed to lay eggs for 2-hour intervals. Embryos were collected, fixed and immuno-stained with mAb MH27 (DSHB, University of Iowa; recognizing AJM-1) and polyclonal LIN-26 antibodies (epidermal nuclei; Labouesse et al, 1996), or with the mAb NE8-4C6 (muscle marker; Schnabel, 1995) and polyclonal 4F2 antibodies (against VAB-10A; Bosher et al , 2003) as described elsewhere (Bosher et al, 2003). The NE8/4C6 monoclonal antibody was provided by the Medical Research Council. Stacks of images every 0.3 µm were captured using a confocal microscope (Leica SP2 AOBS RS); generally 10 confocal sections were projected using ImageJ and then processed using Adobe Photoshop.

For whole worm staining, mix-staged worms were collected and washed extensively several times with M9 buffer and staining was performed as by [45]. Fixed and permeabilized animals were incubated overnight at 4°C with purified rabbit anti-ALG-1 antibody (1∶100) and probed with Alexa Fluor 488 anti-rabbit (1∶500) (Molecular Probes) as secondary antibody for 4 hours at room temperature. Images were captured using Zeiss motorized Axioplan 2 microscope at 630× with an AxioCam MRm camera and AxioVision acquisition software.

### MicroRNA array profiling in ALG-1 and ALG-2 complexes

The transgenic animals expressing integrated arrays of either ALG-1 or GFP::ALG-2 tagged protein were harvested at specific development stages corresponding to four different larval transitions and total protein lysates were prepared as described previously [12]. Protein lysates prepared from *alg-1 (gk214)* and for wild-type N2 animals were used as controls for ALG-1 and GFP::ALG-2, respectively. Immunoprecipitations were performed by preclearing 4 mg of total protein with 20 µl of protein-G magnetic beads (invitrogen) for 1 h at 4°C. The cleared lysates were then incubated for 2 h at 4°C with 20 µL of protein-G magnetic beads conjugated with either 5 µg of affinity-purified polyclonal anti-ALG-1 antibody [14] or with the monoclonal antibody anti-AFP 3E6 (QBiogene). The beads were then washed three times with ice-cold lysis buffer containing 1% of Superasin (Roche). 90% of the purified beads were used for total RNA extraction performed as described in [46]. The remaining 10% of beads were boiled in SDS loading buffer and protein resolved by SDS-PAGE on an 8% gel. To detect ALG-1 and GFP-tagged ALG-2, the membranes were incubated overnight at 4°C with either affinity-purified polyclonal anti-ALG-1 diluted 1∶5000 or a mouse monoclonal anti-GFP (Roche) diluted 1∶2000 in TBST-milk solution, incubated 1 h at room temperature with either anti-rabbit (ALG-1) or anti-mouse (GFP-ALG-2) HRP-conjugated secondary antibody and then visualized by Western Lightening ECL Kit (Perkin Elmer).

RNA molecules extracted from ALG-1 and ALG-2 complexes were then subjected to size selection and purified small RNAs were used for miRNA array profilings as performed in [47]. All miRNA expression data have been submitted to the Gene Expression Omnibus (GEO) with accession number GSE35505 (for microarray platform used).

## Supporting Information

Figure S1 **ALG-1 and ALG-2 expression profiles.** GFP::ALG-2 and RFP::ALG-1 expression in body neurons (BN), seam cells (SC), excretory system cell (X), uterine cells lining the uterine cavity (U) and gonadal sheet cells (GS). Scale bar 20 µm.
(TIF)

Figure S2 **ALG-1 and ALG-2 expression profiles.** GFP::ALG-2 and RFP::ALG-1 expression in head hypodermal cell (HH), tail hypodermal cells and tail neurons (TH,TN), body muscle cells (BM) and larval P cells whose lineage contribute to the neurons and body hypodermis (BH). Scale bar 20 µm.
(TIF)

Figure S3 **Immunostaining of ALG-1 in adult** ***C. elegans*** **hermaphrodites.** Staining with polyclonal antibody against ALG-1 of head organs. (A) A specific signal is detected in the pharynx of wild-type animals. (B) Staining of control *alg-1(gk214)* animal.
(TIF)

Figure S4 **Detection of ALG-1 and GFP::ALG-2 in purified complexes.** Detection of ALG-1 (A) and GFP::ALG-2 (B) by Western blot analysis found in the immunopurified (IP) complexes from each developmental stage used for microRNA microarrays. 50 µg of the total protein (in) were run as controls.
(TIF)

Movie S1 **Time-lapse analysis of the** ***alg-2(ok304)*** **;** ***alg-1(gk214)/+*** **embryos.** Movie from the time-lapse microscopy shown in Figure 7. The double mutant *alg-2; alg-1* embryo is located at the bottom.
(MP4)
