# Supplementary figures and images for: Developmental Characterization of the MicroRNA-Specific C. elegans Argonautes alg-1 and alg-2
Source: PLoS One. 2012 Mar 20;7(3):e33750. doi: 10.1371/journal.pone.0033750 (PMC3309000; doi:10.1371/journal.pone.0033750)

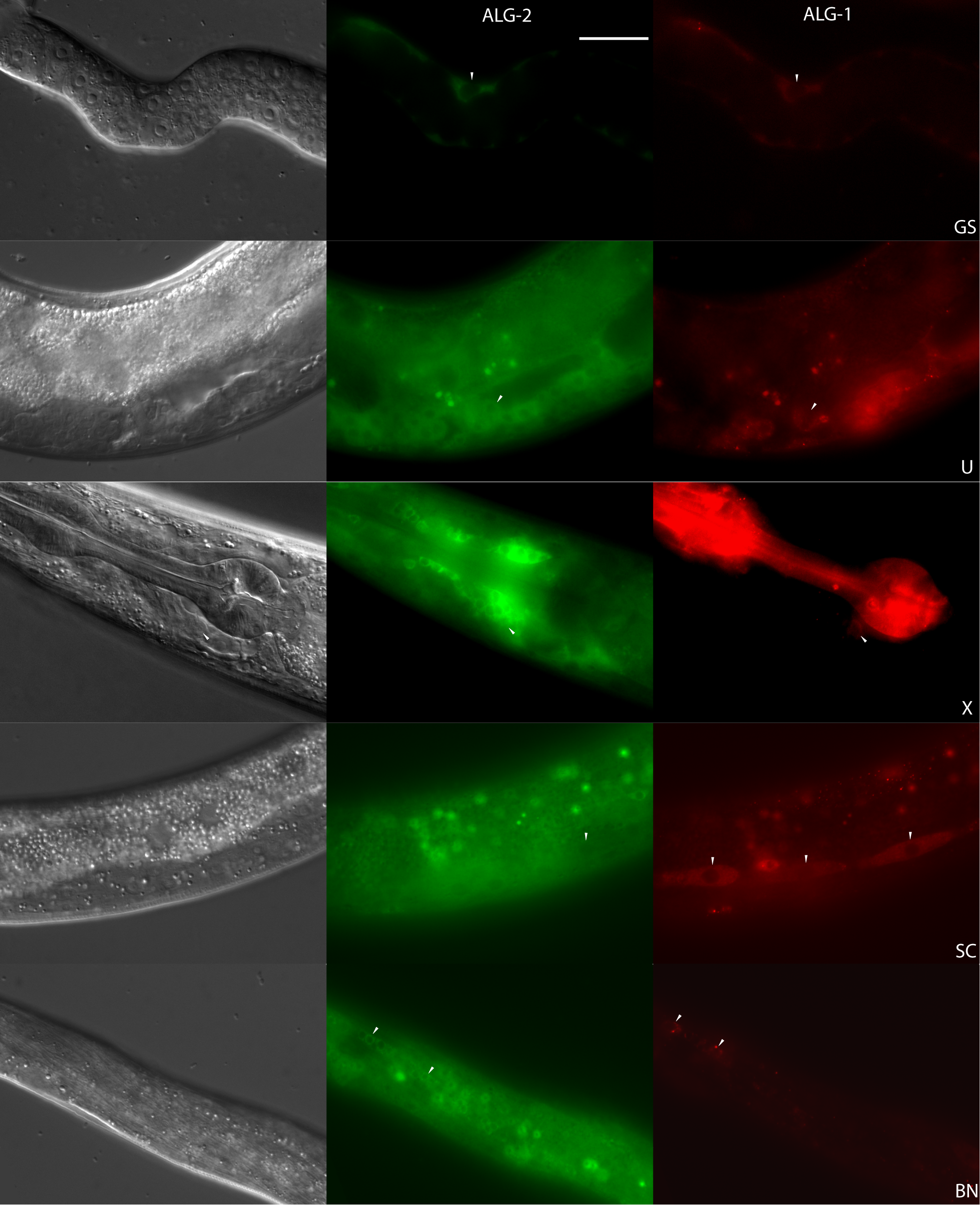

Supplement: Figure S1 — ALG-1 and ALG-2 expression profiles. GFP::ALG-2 and RFP::ALG-1 expression in body neurons (BN), seam cells (SC), excretory system cell (X), uterine cells lining the uterine cavity (U) and gonadal sheet cells (GS). Scale bar 20 µm. (TIF) [file pone.0033750.s001.tif]

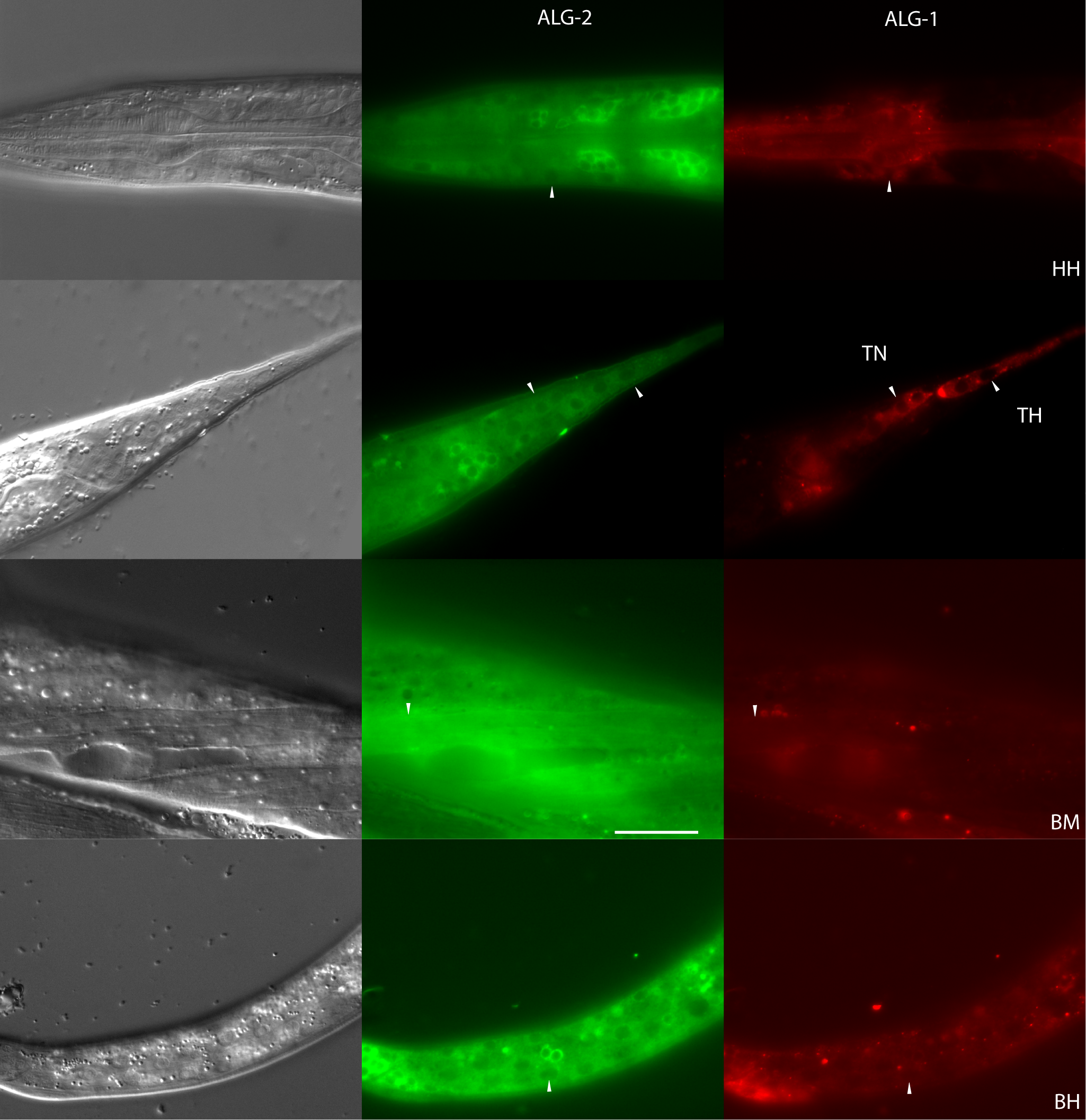

Supplement: Figure S2 — ALG-1 and ALG-2 expression profiles. GFP::ALG-2 and RFP::ALG-1 expression in head hypodermal cell (HH), tail hypodermal cells and tail neurons (TH,TN), body muscle cells (BM) and larval P cells whose lineage contribute to the neurons and body hypodermis (BH). Scale bar 20 µm. (TIF) [file pone.0033750.s002.tif]

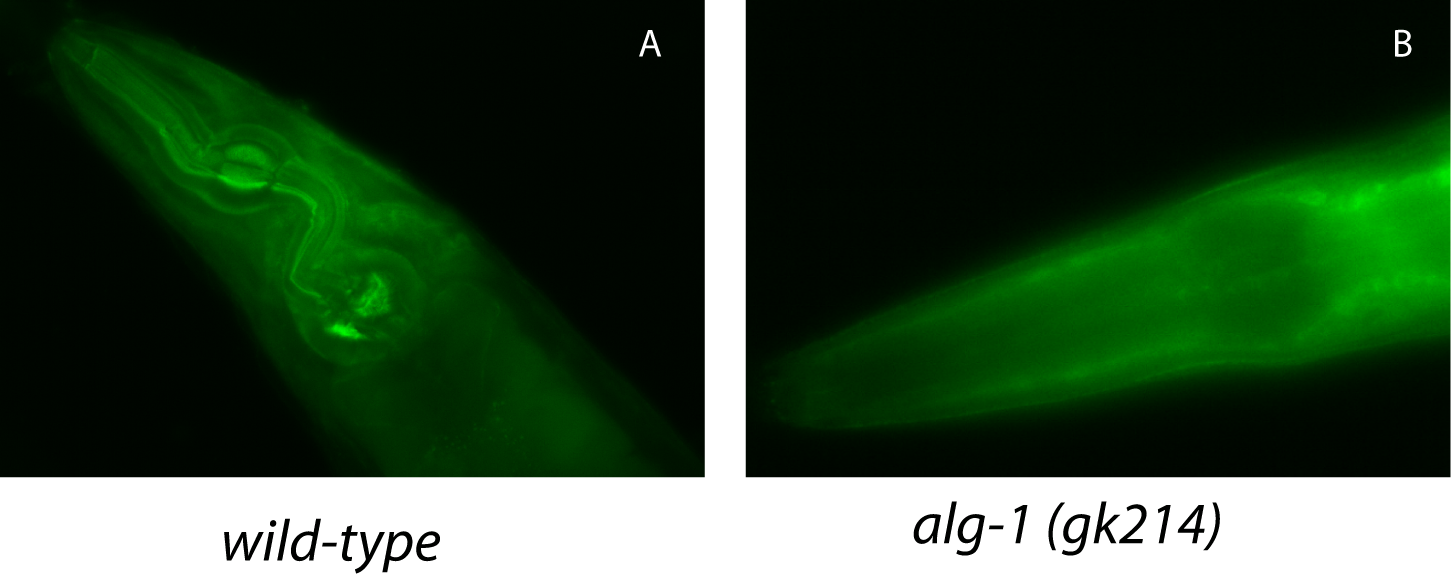

Supplement: Figure S3 — Immunostaining of ALG-1 in adult C. elegans hermaphrodites. Staining with polyclonal antibody against ALG-1 of head organs. (A) A specific signal is detected in the pharynx of wild-type animals. (B) Staining of control alg-1(gk214) animal. (TIF) [file pone.0033750.s003.tif]

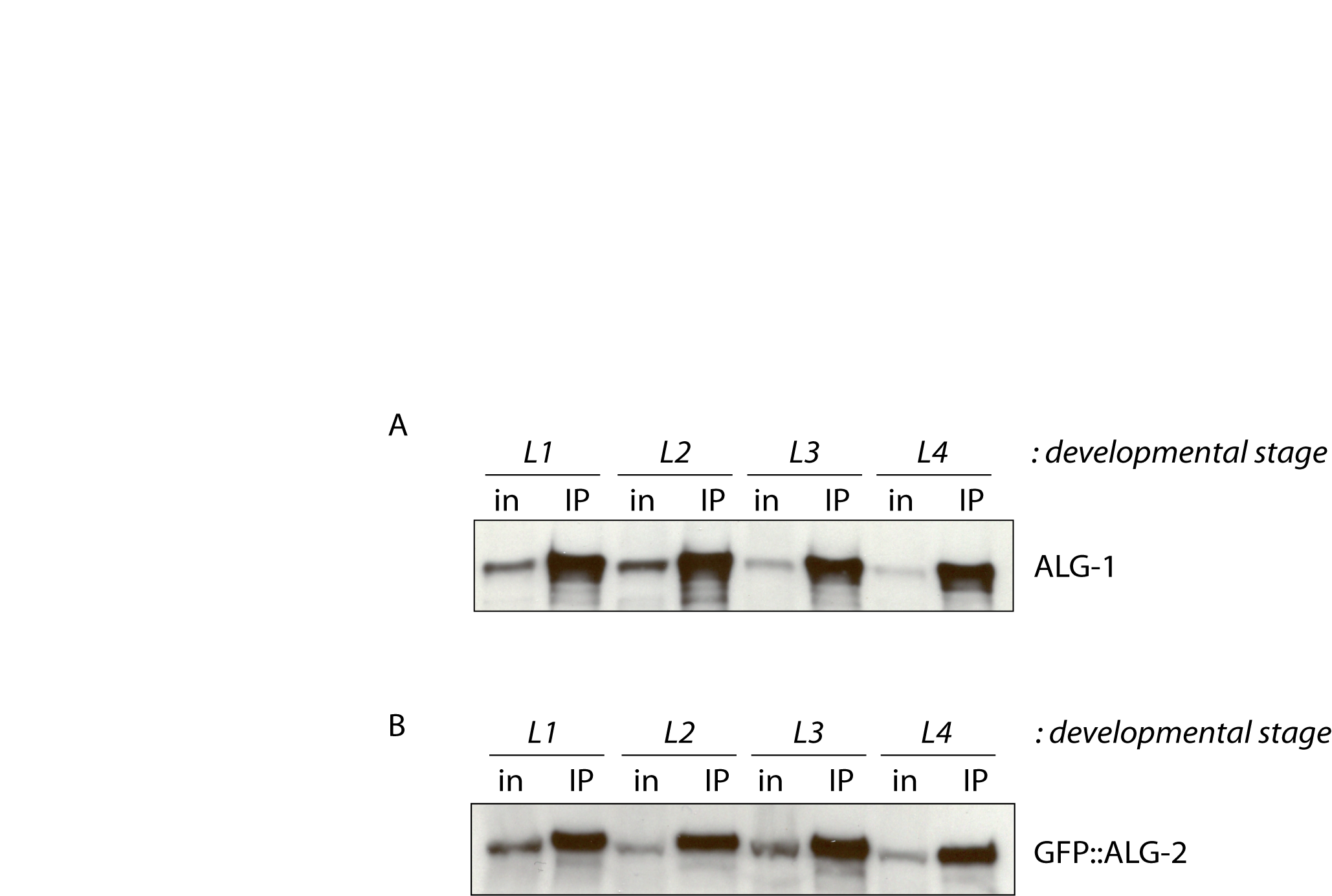

Supplement: Figure S4 — Detection of ALG-1 and GFP::ALG-2 in purified complexes. Detection of ALG-1 (A) and GFP::ALG-2 (B) by Western blot analysis found in the immunopurified (IP) complexes from each developmental stage used for microRNA microarrays. 50 µg of the total protein (in) were run as controls. (TIF) [file pone.0033750.s004.tif]
